# The Role of Fear and Disgust in Predicting the Effectiveness of Television Advertisements That Graphically Depict the Health Harms of Smoking

**DOI:** 10.5888/pcd11.140326

**Published:** 2014-12-11

**Authors:** Harpa Lind Jónsdóttir, Jeffrey E. Holm, Dmitri Poltavski, Nancy Vogeltanz-Holm

**Affiliations:** Author Affiliations: Harpa Lind Jónsdóttir, Jeffrey E. Holm, Dmitri Poltavski, University of North Dakota, Grand Forks, North Dakota.

## Abstract

**Introduction:**

Antismoking television advertisements that depict the graphic health harms of smoking are increasingly considered best practices, as exemplified by the Centers for Disease Control and Prevention’s current national campaign. Evaluation of responses to these widely used advertisements is important to determine advertisements that are most effective and their mechanisms of action. Our study tested the hypothesis that advertisements rated highest in fear- and disgust-eliciting imagery would be rated as the most effective.

**Methods:**

Our laboratory study included 144 women and men aged 18 to 33; 84% were current nonsmokers. All participants viewed 6 antismoking television advertisements that depicted the health harms of smoking; they rated their responses of fear and disgust and the effectiveness of the advertisements. We used multilevel modeling to test the effects of the following in predicting effectiveness: fear, disgust, the fear–disgust interaction, the advertisement, and the participant’s sex and smoking status. Follow-up analyses examined differences in ratings of fear, disgust, and effectiveness.

**Results:**

Advertisement, fear, disgust, and the fear–disgust interaction were each significant predictors of effectiveness. Smoking status and sex were not significant predictors. The 3 advertisements that elicited the highest ratings of fear and disgust were rated the most effective.

**Conclusion:**

Our findings support the hypothesis that antismoking advertisements of health harms that elicit the greatest responses of fear or disgust are the most effective. When advertisements elicit high ratings of both fear and disgust, advertisements with graphic imagery are effective, whereas advertisements without graphic imagery are not.

## Introduction

Cigarette smoking in the United States has decreased in part because of antismoking mass media campaigns (1–7). Subgroup analyses by age, sex, race/ethnicity, socioeconomic status, and smoking status support the concept that advertisements depicting the health harms of smoking, especially those using graphic imagery, are potentially more effective than neutral or positive advertisements (4–6,8–13). Advertisements that graphically depict the health harms of smoking are increasingly considered best practices, as exemplified by the recent antismoking campaign launched by the Centers for Disease Control and Prevention (CDC) (7). Researchers have theorized that the mechanism of action of graphic antismoking advertisements is the elicitation of negative emotional responses, which leads to greater awareness and greater impact, as measured directly by smoking-related behaviors and more often indirectly through recall of advertisements and perceived effectiveness ratings (8–13). Vogeltanz-Holm et al (10) and Leshner et al (14) used cognitive and learning theories to propose further that advertisement effectiveness ratings and recall are greatest when advertisements elicit the emotional responses of fear or disgust or both. Leshner et al, however, suggested that when antismoking advertisements contain high levels of imagery that elicits both fear and disgust, their effectiveness may be reduced. Evaluation of viewers’ emotional and cognitive–intellectual appraisal of graphic advertisements of health harms are needed to further test mechanisms of action and understand advertisements that are most effective for use in state and national campaigns.

The objective of our study was to examine the responses of fear and disgust to 6 antismoking advertisements and the ratings of perceived effectiveness of the advertisements among a sample of young adults. We sought to determine whether fear and disgust were independently or in combination significant predictors of perceived effectiveness. We also sought to examine ratings of the advertisements to elucidate the relationship between advertisement content and perceived effectiveness. Our study tested the hypothesis that advertisements rated highest in fear- and disgust-eliciting imagery would be rated as the most effective. 

## Methods

We recruited a sample of young adults to complete a questionnaire and view 6 antismoking television advertisements in a laboratory setting from January through May 2013. We used multilevel modeling to test the effects of the following in predicting effectiveness: fear, disgust, the fear–disgust interaction, the advertisement, and the participant’s sex and smoking status. Differences in advertisements by ratings of fear, disgust, and perceived effectiveness were analyzed to understand mechanisms of action in effective antismoking health-harms advertisements. The institutional review board at the University of North Dakota approved this study.

### Study sample

Participants (N = 144) were women (n = 86) and men (n = 58) aged 18 to 33 years (mean, 19.8 y; standard deviation, 2.0 y) enrolled in a Midwestern university and who received research credit for their participation. To be eligible for the study, participants had to be aged 18 or older and enrolled in an undergraduate psychology course. All participants were recruited using the SONA research management system (Sona Systems Ltd), and all provided informed consent. Most participants reported their race/ethnicity as non-Hispanic white (92%). The remaining participants were Asian (4%), Hispanic (1.5%), American Indian (1.5%), and African American (1%). Power analyses using G*Power 3.1 (15) and setting α at .05 and power at 0.8 indicated that the sample size was sufficient to detect small effects in the multilevel analyses and the within-subject analyses of variance (ANOVAs).

### Smoking status of study participants

Participants first completed a demographic questionnaire that included questions about tobacco use; participants who self-reported as current smokers then completed the Fagerström Test for Nicotine Dependence (16). These data showed that most participants (n = 121) were not current smokers, and of those that were current smokers (n = 23), 22 reported smoking fewer than 5 cigarettes per day. Scores on the Fagerström scale (range, 0 to 10) showed that 78.3% of smokers had very low nicotine dependence (scores of 0 to 2) and 21.7% had low nicotine dependence (scores of 3 or 4).

### The 6 advertisements selected for study

Six advertisements were selected from the CDC’s Media Campaign Resource Center (17). All 6 advertisements were selected because they had been evaluated in previous state or national antismoking media campaigns and identified in studies (9,10,14) as having health-harms content that varied in graphic imagery. Two advertisements (“Terrie’s Tip” and “Suzy’s Tip”) were part of CDC’s recent 3-month campaign, Tips from Former Smokers. The 2 new CDC advertisements have not been evaluated for emotional response or perceived effectiveness among young adults. All advertisements were approximately 30 seconds in duration and of high production quality. 

The advertisement “Artery” first aired in an Australian national campaign in 2000. It shows a physician removing fatty deposits from the aorta of a 32-year-old deceased smoker ( http://nccd.cdc.gov/MCRC/Apps/SearchDetails.aspx?CatalogID=198&IFS=11336 ). The advertisement “Brain” was from the same Australian campaign and shows a brain being cut in half to show a blood clot formed by smoking ( http://nccd.cdc.gov/MCRC/Apps/SearchDetails.aspx?CatalogID=741&IFS=17090 ). The advertisement “Echo” was part of a California state campaign in 2002. It shows several people discussing why they cannot quit smoking; each person gives an excuse, and between each excuse, a person either sick or dying from tobacco use provides an ironic analogy to the excuse ( http://nccd.cdc.gov/MCRC/Apps/SearchDetails.aspx?CatalogID=986&IFS=21542 ). The advertisement “Still Can’t Quit” shows a teenaged boy in a hospital room explaining that he has spots on his lung but he still cannot quit smoking. It was part of an Iowa state campaign in 2002 ( http://nccd.cdc.gov/MCRC/Apps/SearchDetails.aspx?CatalogID=925&IFS=17810 ). The advertisements “Suzy’s Tip” and “Terrie’s Tip” were part of the CDC campaign that first aired in 2012. “Suzy’s Tip” shows Suzy talking about losing her independence after smoking caused her to have a stroke while her son gives her a sponge bath ( http://nccd.cdc.gov/MCRC/Apps/SearchDetails.aspx?CatalogID=2186&IFS=55750 ). “Terrie’s Tip” shows Terrie getting ready for the day after the effects of treatments of throat cancer caused her to lose her teeth and hair and to have a tracheotomy ( http://nccd.cdc.gov/MCRC/Apps/SearchDetails.aspx?CatalogID=2187&IFS=33368 ).

### Measures

Participants viewed all 6 advertisements in random order on a computer in a laboratory. After viewing each advertisement, each participant responded to the following 5 fear- and disgust-related adjectives used in a previous study (14): “frightening,” “scary,” “sickening,” “repulsive,” and “gross*.*” For each adjective, participants circled a number on a 5-point Likert scale that included the anchors “1 = Not at all” and “5 = Very much so.” For each advertisement, participants then responded to 4 measures of perceived effectiveness 1) “This ad had a message that was important to me,” 2) “This ad made me stop and think about my health,” 3) “This ad is one that I will likely tell other people about,” and 4) “Overall I thought this ad was a very good antismoking ad.” For each measure, participants responded by circling a number on a 5-point Likert scale (1 = Strongly disagree, 2 = Disagree, 3 = Neither disagree or agree, 4 = Agree, and 5 = Strongly agree). These perceived effectiveness measures were used in previous studies (8,10,18).

### Data analysis

Composite measures of fear, disgust, and perceived effectiveness were created by examining factor analytic models and conducting reliability analyses to determine internal consistency estimates. These analyses supported the construction of the following 3 composite measures: 1) a measure of fear was created by averaging together the ratings of “frightening” and “scary” (Cronbach α = 0.94); 2) a measure of disgust was created by averaging together the ratings of “sickening,” “repulsive,” and “gross” (Cronbach α = 0.94); and 3) a measure of perceived effectiveness was created by averaging together all ratings of perceived effectiveness (Cronbach α = 0.92).

SPSS 22 (IBM Corporation) was used to conduct all analyses. Ratings of fear and disgust were entered into a multilevel model as fixed effects along with advertisement, sex of participant, and smoking status of participant to determine the relative importance of fear and disgust in predicting participants’ ratings of perceived effectiveness. Fear and disgust were allowed to interact in this analysis. Data on participants were nested within advertisement in this model because all participants viewed all advertisements. A compound symmetry covariance matrix was selected using the Akaike Information Criterion adequacy-of-fit measure.

When we found predictors of perceived effectiveness, we conducted 2 additional sets of analyses for each of the 6 advertisements. We examined bivariate correlations between perceived effectiveness and fear, disgust, the fear–disgust interaction, and mean centering of the fear–disgust interaction. Mean centering of the fear–disgust interaction permitted us to compare the effect of the interaction with average disgust and fear responses across all advertisements. We also conducted repeated-measures ANOVAs adjusted for sphericity to examine differences in fear responses, disgust responses, the fear–disgust interaction, and ratings of perceived effectiveness. Bonferroni-adjusted post hoc tests were used to control for type I error increases associated with multiple comparisons.

## Results

The multilevel analysis showed significant main effects for fear (*F*_1,819 _= 115.2, *P* < .001), disgust (*F*_1,794_ = 21.7, *P* < .001), the fear–disgust interaction (*F*_1,778_ = 11.2, *P* = .001), and advertisement (*F*_5,735_ = 3.34, *P* = .005). The effects of sex and smoking status were not significant. Parameter estimates indicate that fear and disgust and their interaction each had a significant independent effect on ratings of perceived effectiveness (Table 1). After accounting for the effects of fear and disgust, differences in ratings of perceived effectiveness were significant for 2 advertisements, “Terrie’s Tip” and “Artery” (Table 1).

**Table 1:** Multilevel Model for Predicting Perceived Effectiveness Ratings Among Study Participants (N = 144) for 6 Antismoking Television Advertisements Depicting Health Harms, 2013

Model Predictor	Estimate (95% CI)	*t*	*df*	*P* Value
**Intercept**	1.92 (1.66 to 2.18)	14.36	491	<.001
**Participant sex**				
Female	0.01 (−0.20 to 0.22)	0.13	143	.90
Male	0^a^	—	—	—
**Current smoking status of participant**				
Smoker	−0.11 (−0.39 to 0.17)	−0.78	141	.43
Nonsmoker	0^a^	—	—	—
**Advertisement**				
“Brain”	0.10 (−0.05 to 0.24)	1.32	777	.19
“Artery”	0.25 (0.09 to 0.40)	3.13	786	.002
“Terrie’s Tip”	0.23 (0.08 to 0.37)	3.09	787	.002
“Suzy’s Tip”	0.05 (−0.08 to 0.17)	0.75	725	.45
“Still Can’t Quit”	0.05 (−0.07 to 0.17)	0.76	725	.44
“Echo”	0^a^	—	—	—
**Ratings**				
Fear response	0.41 (0.34 to 0.49)	10.73	819	<.001
Disgust response	0.20 (0.11 to 0.28)	4.66	794	<.001
Fear × disgust interaction	−0.04 (−0.06 to 0.02)	−3.34	778	.001

Abbreviations: CI, confidence interval; *df*, degrees of freedom.

a This parameter was set to 0 because it is redundant.

Fear and disgust individually and combined positively predicted ratings of perceived effectiveness for all 6 advertisements (Table 2); however, the centered fear–disgust interaction showed negative relationships in 3 advertisements (“Echo,” “Still Can’t Quit,” and “Suzy’s Tip”). These findings help explain the significant, but negative, effect of the fear–disgust interaction in the multilevel model. Participants who had above-average ratings of both fear and disgust for “Echo,” “Still Can’t Quit,” and “Suzy’s Tip” were more likely to have lower ratings of perceived effectiveness for those advertisements than did those who had above-average ratings of fear *or* disgust. Significant repeated measures ANOVAs (η^2^ ranging from 0.21 to 0.59) showed that the highest ratings of fear were associated with the advertisement “Terrie’s Tip,” whereas the highest ratings of disgust were associated with “Artery” (Table 3). Similarly, in these analyses, the fear–disgust interaction was greatest and the ratings of perceived effectiveness were highest for “Terrie’s Tip,” “Artery,” and “Brain.”

**Table 2:** Bivariate Pearson *r* Correlations Between Perceived Effectiveness Ratings for 6 Antismoking Television Advertisements Depicting Health Harms and Ratings of Fear, Disgust, and Fear × Disgust Among Study Participants (N = 144), 2013

Advertisement	Fear Ratings		Disgust Ratings		Fear × Disgust Interaction		Centered Fear × Disgust Interaction^a^	
	*r*	*P *Value	*r*	*P* Value	*r*	*P *Value	*r*	*P *Value
“Echo”	0.56	<.001	0.26	.002	0.41	<.001	−0.46	<.001
“Still Can’t Quit”	0.58	<.001	0.32	<.001	0.49	<.001	−0.26	.002
“Suzy’s Tip”	0.47	<.001	0.28	<.001	0.39	<.001	−0.26	.002
“Terrie’s Tip”	0.42	<.001	0.23	.006	0.35	<.001	0.16	.049
“Artery”	0.50	<.001	0.30	<.001	0.50	<.001	0.41	<.001
“Brain”	0.41	<.001	0.33	<.001	0.44	<.001	0.25	.003

a The centered interaction subtracts the mean value from each component of the interaction before deriving the product [(Disgust − Mean Disgust) × (Fear − Mean Fear)]. Mean centering of the fear–disgust interaction permitted us to compare the effect of the interaction with average disgust and fear responses across all advertisements.

**Table 3:** Ratings (Mean [SD]) of Fear, Disgust, Fear × Disgust, and Perceived Effectiveness for 6 Antismoking Television Advertisements Depicting Health Harms Among Study Participants (N = 144), 2013

Rating	“Echo”	“Still Can’t Quit”	“Suzy’s Tip”	“Terrie’s Tip”	“Artery”	“Brain”
Fear	2.29^a^ (1.16)	3.01^c^ (1.29)	2.61^b^ (1.29)	3.66^d^ (1.25)	3.05^c^ (1.33)	3.23^c^ (1.22)
Disgust	1.69^a^ (0.89)	1.87^a^ (1.03)	2.40^b^ (1.21)	3.36^c^ (1.23)	3.99^d^ (1.10)	3.49^c^ (1.24)
Fear × disgust	4.42^a^ (4.21)	6.29^b^ (5.35)	6.87^b^ (5.60)	13.05^c^ (7.42)	12.96^c^ (7.27)	11.92^c^ (6.92)
Perceived effectiveness	3.01^a^ (0.94)	3.32^b^ (0.91)	3.23^a^^, ^ b (0.85)	3.80^d^ (0.78)	3.70^c^^, ^ d (0.88)	3.56^c^ (0.79)

Abbreviation: SD, standard deviation.

a–d Means in the same row with the same superscript were not significantly different using Bonferroni-adjusted contrasts.

## Discussion

This study is the first 1) to examine young adults’ emotional responses to 2 new CDC antismoking advertisements and to determine these young adults’ ratings of the advertisements’ perceived effectiveness and 2) to compare these advertisements with 4 other antismoking advertisements previously shown to elicit feelings of fear and disgust. 

Multilevel analyses showed that the greater the response of fear and/or disgust to the 6 advertisements in this study, the greater the ratings of perceived effectiveness; however, the fear–disgust interaction was negatively related to their perceived effectiveness. This finding could be interpreted as suggesting that advertisements rated high in both fear- and disgust-eliciting content, such as “Artery,” are less effective than those rated high only in fear, such as “Still Can’t Quit” (14). However, our follow-up analyses suggest that the relationship between the combination of fear and disgust and perceived effectiveness is mediated by the presence or absence of unambiguous, graphic imagery (ie, diseased body parts or disfigurement resulting from smoking) in the advertisement. “Echo,” “Still Can’t Quit,” and “Suzy’s Tip,” the 3 advertisements for which we found negative relationships between the fear–disgust interaction and perceived effectiveness, did not contain such graphic imagery. In contrast, “Terrie’s Tip,” “Artery,” and “Brain,” each of which demonstrated a positive relationship between the fear–disgust interaction and perceived effectiveness, did contain such graphic imagery. The presence of unambiguous, graphic imagery that elicits responses of fear and disgust, such as that found in “Terrie’s Tip,” “Artery,” and “Brain,” appears to be critical for the fear–disgust interaction to predict greater perceived effectiveness. The content of advertisements such as “Echo,” “Still Can’t Quit,” and “Suzy’s Tip,” which lack such graphic imagery, may be sufficiently ambiguous that individuals who respond to them with high levels of fear and disgust are upset in ways not directly related to smoking.

Further examination of the mean responses to each of the 6 advertisements shows that our findings are consistent with and support previous findings that advertisements eliciting high levels of fear and disgust are correspondingly perceived as most effective (8–13). In contrast to the findings of one study (14), we found that mean ratings of perceived effectiveness mirrored mean scores of the fear–disgust interaction for the advertisement “Terrie’s Tip” (which rated highest in both measures among the 6 advertisements) followed by “Artery,” “Brain,” “Suzy’s Tip,” Still Can’t Quit,” and “Echo” (in that order). This pattern provides a convincing argument for including advertisements depicting graphic health harms of smoking that elicit both high disgust and fear responses. 

Two advertisements, “Terrie’s Tip” and “Suzy’s Tip,” were part of CDC’s recent campaign, Tips from Former Smokers, which resulted in significant increases in the number of quit attempts, nonsmoker quit recommendations, and family/friend discussions about the dangers of smoking (7). In our study, “Terrie’s Tip” had one of the 2 highest ratings of perceived effectiveness, the highest mean rating of fear, and the second-highest mean rating of disgust. Therefore, our study provides further evidence of the overall effectiveness of the advertisement “Terrie’s Tip” and further support for the idea that the advertisement’s effectiveness is probably derived from its ability to elicit both fear and disgust responses.

Overall, our study’s results are consistent with those of most previous studies finding that responses of fear and disgust to antismoking advertisements are important factors in perceptions of effectiveness (8–13). Moreover, our results suggest that the relationship between fear and disgust and perceived effectiveness is determined by whether an advertisement contains unambiguous, graphic images depicting smoking-related health harms that elicit fear and disgust. “Terrie’s Tip,” “Artery,” and “Brain” were the most effective advertisements in our study, the advertisements that most clearly contained such graphic imagery, and the advertisements that showed a positive relationship between the fear–disgust interaction and perceived effectiveness. In contrast, “Echo,” “Still Can’t Quit,” and “Suzy’s Tip” were perceived as less effective, did not contain unambiguous graphic imagery of smoking-related health harms, and showed a negative relationship between the fear–disgust interaction and perceived effectiveness.

Our study has several limitations. Only 16% of our sample self-reported as smokers, and they self-reported almost exclusively as light smokers with low levels of nicotine dependence. More frequent and more dependent smokers might perceive antismoking advertisements differently than the smokers in our study. Participants viewed the advertisements in a laboratory, a different context from that in which antismoking television advertisements are typically viewed. However, field studies (9,10) also find that negative emotional responses such as fear and disgust predict effectiveness. We measured only emotional responses and perceived effectiveness; measuring recall or psychophysiological responses to the advertisements might produce different results.

Despite these limitations, our results can inform public health decisions about advertisements that are most likely to be effective in antismoking media campaigns. Our results also strengthen the hypothesis that the most effective antismoking advertisements are those with graphic images designed to elicit responses of fear or disgust. Eliciting such responses can lead to stronger and more sustained learning of avoidance and cessation of smoking behaviors.

Future studies with larger sample sizes should examine important questions not addressed in our study: how do smoking status, sex, and individual differences, such as depression, affect participant ratings? Also important will be to measure how viewers are attending to, processing, and responding to antismoking television advertisements. Incorporating visual tracking and perceptual measurements, recall or memory tasks, and cortical and autonomic nervous system assessment are all means by which we might further understand how to improve the effectiveness of antismoking television advertisements.
